# Efficacy and safety of PD-1 inhibitors plus chemotherapy with or without endostatin for stage IV lung squamous cancer: a retrospective study

**DOI:** 10.3389/fimmu.2024.1413204

**Published:** 2024-06-07

**Authors:** Chengliu Lv, Yahua Wu, Weiwei Gu, Bin Du, Na Yao, Yingjiao Zhu, Jianping Zheng, Yaping Hong, Jinhuo Lai

**Affiliations:** ^1^ Department of Medical Oncology, Fujian Medical University Union Hospital, Fuzhou, Fujian, China; ^2^ Department of Medical Oncology, People’s Hospital Affiliated to Shandong First Medical University, Jinan, Shandong, China; ^3^ Department of Medical Oncology, Shengli Clinical Medical College, Fujian Medical University, Fujian Provincial Hospital, Fuzhou, Fujian, China; ^4^ Department of Medical Oncology, Fujian Medical University Cancer Hospital, Fuzhou, Fujian, China

**Keywords:** lung squamous cell carcinoma, PD-1 inhibitor, chemotherapy, endostatin, lung immune prognostic index

## Abstract

**Backgroud:**

The study aimed to analyze the efficacy and safety of PD-1 inhibitors plus chemotherapy with or without endostatin for stage IV lung squamous cell carcinoma (LUSC).

**Methods:**

A total of 219 patients with stage IV LUSC were included. 120 received PD-1 inhibitors plus chemotherapy with or without endostatin (IC ± A), of which 39 received endostatin (IC+A) and 81 did not receive endostatin (IC-A). 99 received chemotherapy with or without endostatin (C ± A). Endpoints included overall survival (OS), progression-free survival (PFS), adverse events (AEs), and immune-related adverse events (irAEs).

**Results:**

The median PFS in the IC ± A group versus the C ± A group was 8 and 4 months (P < 0.001), and the median OS was 17 and 9 months (P < 0.001). There was no significant difference in any grade AEs between the IC ± A and C ± A groups (P > 0.05). The median PFS in the IC+A group versus the IC-A group was 11 and 7 months (P = 0.024), and the median OS was 34 and 15 months (P = 0.01). There was no significant difference between the IC+A group and the IC-A group for all grade AEs and irAEs (P > 0.05). The subgroup analysis showed that patients with LIPI = 0 had significant OS and PFS benefits in IC+A group, while for patients with LIPI = 1–2, there was no significant difference in OS and PFS benefits between the IC+A group and IC-A group.

**Conclusions:**

PD-1 inhibitors plus chemotherapy with endostatin might be first-line treatment for patients with stage IV LUSC.

## 1 Introduction

Stage IV lung squamous cell carcinoma (LUSC) typically carries a poor prognosis (1, 2). However, survival rates among LUSC patients have improved with the application of immune checkpoint inhibitors (ICIs), particularly PD-1 inhibitors. Consequent to data unearthed from several clinical studies, the combination of PD-1 inhibitors with chemotherapy has emerged as the standard first-line treatment for stage IV driver gene-negative LUSC (3–6). While this combination enhances survival rates in patients with advanced LUSC, the emergence of drug resistance remains a critical concern, limiting the potential benefits (7, 8).

Endostatin (recombinant human vascular endostatin), targeting the endothelial cells of tumor vasculature, inhibits neovascularization, thereby impeding nutrient supply to tumor cells and curbing their proliferation and metastasis (9). As an anti-angiogenic agent, endostatin influences the tumor immune microenvironment similarly to PD-1 inhibitors, providing a rationale for their concurrent use (9, 10). A retrospective clinical study has demonstrated that the combination of ICIs with endostatin offers greater efficacy and safety than the combination of ICIs with chemotherapy in treating advanced non-small cell lung cancer (NSCLC) (11). Furthermore, Phase II clinical trials in the Lung-MAP S1800A study have shown that combining pembrolizumab with ramucirumab leads to improved efficacy and survival outcomes for patients with advanced LUSC (12).

Our research focuses on determining the potential of endostatin to enhance the efficacy of PD-1 inhibitors in conjunction with chemotherapy for treating stage IV LUSC. Given the insufficient clinical evidence to support the combined usage of PD-1 inhibitors, chemotherapy, and endostatin, our study is designed to evaluate the efficacy and safety of this treatment regimen. Specifically, we aim to elucidate endostatin’s impact on the outcome and adverse effects when simultaneously administered with PD-1 inhibitors and chemotherapy in stage IV LUSC patients.

## 2 Methods

### 2.1 Patients

This retrospective study included patients diagnosed with stage IV LUSC at our hospital from 2018 to 2023 who were first-line received PD-1 inhibitor combined with chemotherapy or chemotherapy alone with or without endostatin therapy. Inclusion criteria: (1) pathological diagnosis was LUSC; (2) clinical stage was stage IV; (3) Eastern Cooperative Oncology Group Performance Status (ECOG PS) was 0–2. Exclusion criteria: (1) patients’ age less 18 years or over 85 years; (2) patients with other primary malignancies; (3) lack of clinical hematological and imaging data. All patients were clinically staged using the 8th edition of the American Joint Committee on Cancer (AJCC) TNM staging system. According to the inclusion and exclusion criteria, 219 patients with stage IV LUSC were finally enrolled.

### 2.2 Data collections

Clinical data included baseline data before receiving anti-tumor therapy: gender, age, ECOG PS, smoking history, histological type, clinical stage, distant metastatic(brain, liver, bone), PD-L1 expression level, and Lung Immune Prognostic Index (LIPI). LIPI is based on the derived neutrophil to lymphocyte ratio (dNLR) and lactate dehydrogenase (LDH), dNLR = baseline neutrophil count/(white blood cell-neutrophil count), and is calculated as 1 point for dNLR greater than 3 or LDH greater than normal. Patients are divided into two groups with good (0 points) and poor (1–2 points) prognosis (13, 14). Other relevant clinical data: anti-tumor drugs, chemotherapy cycle, survival events, treatment efficacy, AEs and irAEs.

### 2.3 Treatment regimen

IC ± A group received PD-1 inhibitor plus chemotherapy with or without endostatin, IC + A group received PD-1 inhibitor plus chemotherapy plus endostatin, IC-A group received PD-1 inhibitor plus chemotherapy without endostatin, C ± A group received chemotherapy with or without endostatin. PD-1 inhibitors: pembrolizumab(200 mg iv q3w d1) or sintilimab (200 mg iv q3w d1) or camrelizumab (200 mg iv q3w d1) or tislelizumab (200 mg iv q3w d1). Chemotherapy: paclitaxel (175 mg/m2 iv q3w d1) plus carboplatin (400 mg/m2 iv q3w d1) or cisplatin (100 mg/m2 iv q3w d1). Endostatin:(15 mg qd iv q3w d0–6) was given intravenously at a dose of 15 mg for 3 hours once daily for 7 days. All patients received two cycles of treatment at least.

### 2.4 Outcomes

Overall survival (OS) is defined as the time between the first treatment and death from any cause or the last follow-up. Progression-free survival (PFS) is the time from the first treatment to disease progression, death from any cause, or the last follow-up. Objective response rate (ORR) is defined as the proportion of patients who achieve complete response (CR) or partial response (PR). Disease control rate (DCR) is defined as the proportion of patients who achieve CR, PR, and stable disease (SD). RECIST1.1 solid tumor evaluation criteria were used for short-term efficacy evaluation. All patients were followed up until September 2023.

### 2.5 Adverse events

Adverse events (AEs) and immune-related adverse events (irAEs) occurred during treatment were collected through the medical record system. AEs included anemia, neutropenia, thrombocytopenia, alanine aminotransferase(ALT) elevation, creatinine elevation, nausea, vomiting, decreased appetite, bronchial or pulmonary infection, rash, diarrhea, pain, and insomnia. irAEs included hyperthyroidism, hypothyroidism, adrenocortical insufficiency, pneumonia, severe skin reaction, hepatitis, nephritis, colitis, myocarditis, hypophysitis, pancreatitis, arthritis, peripheral neuropathy, and cardiac arrhythmias. All adverse events were graded according to the National Cancer Institute Common Terminology Criteria for Adverse Events (CTCAE version 5.0).

### 2.6 Statistical analysis

We used Pearson’s chi-square test or Fisher’s exact test to compare categorical variables. The Kaplan-Meier method was used to plot survival curves, and the log-rank test was used for differences between survival curves. Variables with a P value ≤ 0.05 in univariate Cox analysis were included in the multivariate Cox analysis. Multivariate Cox analysis was used to determine independent prognostic factors affecting OS and PFS. Therefore, a statistical result P value < 0.05 was considered statistically significant. All analyses were performed using SPSS 25.0 (IBM, Armonk, NY, USA) for all of the above statistical analyses.

## 3 Result

### 3.1 Patient characteristics

A total of 219 patients with stage IV LUSC were enrolled in our study. The baseline characteristics are shown in **Tables 1**, **2**. There were 120 patients in the IC ± A group and 99 patients in the C ± A group. There were 39 patients in the IC+A group and 81 patients in the IC-A group. The IC ± A group and the C ± A group, the IC+A group and the IC-A group were mostly male, ECOG PS 0–1, smoking history, stage IVA, no brain metastasis, no liver metastasis, no bone metastasis, and the chemotherapy cycle ≥ 4. Except for the PD-L1 expression status, there were no statistical differences in other baseline characteristics between the IC ± A group and the C ± A group. There was no statistical difference in baseline characteristics between the IC+A group and the IC-A group.

**Table 1:** Baseline characteristics of IC ± A and C ± A.

Characteristics	IC ± A group (n=120)	C ± A group (n=99)	P value
Age ≤65 >65	76 (63.3%) 44 (36.7%)	56 (56.6%) 43 (43.4%)	0.308
Gender Male Female	112 (93.3%) 8 (6.7%)	90 (90.9%) 9 (9.1%)	0.505
ECOG PS 0-1 2	103 (85.8%) 17 (14.2%)	84 (84.8%) 15 (15.2%)	0.837
Smoking history Yes No	99 (82.5%) 21 (17.5%)	80 (80.8%) 19 (19.2%)	0.747
PD-L1 Negative 1-49% ≥50% Unknown	26 (21.7%) 31 (25.8%) 13 (10.8%) 50 (41.7%)	15 (15.2%) 6 (6.1%) 4 (4%) 74 (74.7%)	< 0.001
Clinical stage IVA IVB	99 (82.5%) 21 (17.5%)	78 (78.8%) 21 (21.2%)	0.487
Brain metastases No Yes	117 (97.5%) 3 (2.5%)	94 (94.9%) 5 (5.1%)	0.523
Liver metastases No Yes	106 (88.3%) 14 (11.7%)	88 (88.9%) 11 (11.1%)	0.898
Bone metastases No Yes	95 (79.2%) 25 (20.8%)	82 (82.8%) 17 (17.2%)	0.493
Endostatin therapy Yes No	39 (32.5%) 81 (67.5%)	33 (33.3%) 66 (66.7%)	0.896
Chemotherapy cycle 2-3 ≥4	19 (15.8%) 101 (84.2%)	21 (21.2%) 78 (78.8%)	0.305
LIPI 0 1-2	69 (57.5%) 51 (42.5%)	53 (53.5%) 46 (46.5%)	0.557

**Table 2:** **Baseline characteristics of IC+A and IC-A**.

Characteristics	IC+A group (n=39)	IC-A group (n=81)	P value
Age			0.082
≤65 >65	29 (74.4%) 10 (25.6%)	47 (58%) 34 (42%)	
Gender			0.714
Male Female	36 (92.3%) 3 (7.7%)	76 (93.8%) 5 (6.2%)	
ECOG PS			0.769
0-1 2	34 (87.2%) 5 (12.8%)	69 (85.2%) 12 (14.8%)	
Smoking history			0.147
Yes No	35 (89.7%) 4 (10.3%)	64 (79%) 17 (21%)	
PD-L1			0.206
Negative 1-49% ≥50% Unknown	7 (17.9%) 12(30.9%) 7 (17.9%) 13 (33.3%)	19 (23.5%) 19(23.5%) 6 (7.4%) 37 (45.6%)	
Clinical stage			0.147
IVA IVB	35(89.7%) 4 (10.3%)	64 (79%) 17 (21%)	
Brain metastases			0.553
No Yes	39(100%) 0 (0%)	78 (96.3%) 3 (3.7%)	
Liver metastases			0.524
No Yes	36 (92.3%) 3 (7.7%)	70 (86.4%) 11 (13.6%)	
Bone metastases			0.308
No Yes	33 (84.6%) 6 (15.4%)	62 (76.5%) 19 (23.5%)	
Chemotherapy cycle			0.660
2-3 ≥4	7 (17.9%) 32 (82.1%)	12 (14.8%) 69 (85.2%)	
LIPI			0.310
0 1-2	25 (64.1%) 14 (35.9%)	44 (54.3%) 37 (45.7%)	
PD-1 inhibitors			0.938
Pembrolizumab Tislelizumab Sintilimab Camrelizumab	12(30.8%) 9(23.1%) 11(28.2%) 7(17.9%)	29(35.8%) 17(21.0%) 23(28.4%) 12(14.8%)	
Chemotherapy			0.692
Carboplatin Cisplatin	26(66.7%) 13(33.4%)	51(62.9%) 30(37.1%)	

### 3.2 Outcome and efficacy analysis

**Figure 1:**
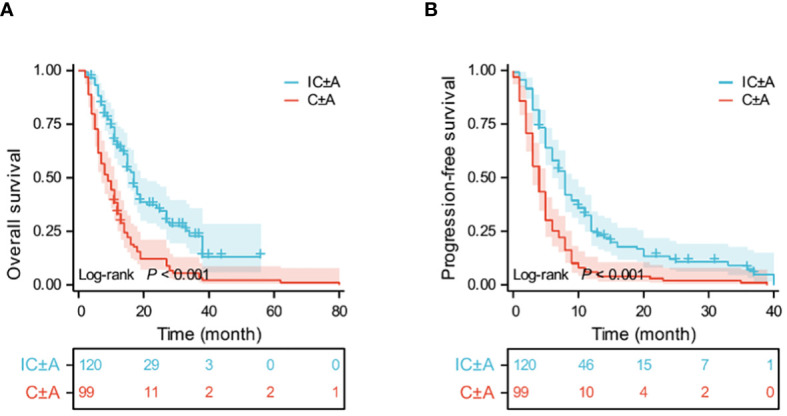
Kaplan-Meier estimates of OS **(A)** and PFS **(B)** in the IC ± A and C ± A population; IC ± A, PD-1 inhibitor plus chemotherapy with or without endostatin; C ± A, chemotherapy with or without endostatin; OS, overall survival; PFS, progression-free survival; Time, month.

Until September 2023, 105 PFS events (87.5%) and 76 OS events (63.4%) occurred in the IC ± A group and 99 PFS events (100%) and 96 OS events (97%) occurred in the C ± A group. The median OS was 17 (95% CI: 15–19) and 9 (95% CI: 6.6–11.4) months (P < 0.001, **Figure 1A**), and the median PFS of the IC ± A group and the C ± A group was 8 (95% CI: 6.9–9.1) and 4 (95% CI: 3.2–4.8) months respectively (P < 0.001, **Figure 1B**). The IC ± A group had longer median PFS and median OS than the C ± A group. In addition, 2 patients (1.6%) achieved CR, 57 patients (47.5%) achieved PR, 44 patients (36.7%) achieved SD, and 17 patients (14.2%) achieved PD in the IC ± A group, with an ORR of 49.2% and a DCR of 85.8%. In the C ± A group, 33 patients (30.3%) achieved PR, 45 patients (45.5%) achieved SD, and 29 patients (29.3%) achieved PD, with an ORR of 25.3% and a DCR of 70.7% (**Table 3**). The ORR (P < 0.001) and DCR (P = 0.006) were better in the IC ± A group than in the C ± A group.

**Table 3:** Evaluation of outcomes in 219 patients with Stage IV LUSC.

Characteristics	IC ± A (n=120)	C ± A (n=99)	P value
Best response, n (%)			0.001
CR	2 (1.7%)	0 (0%)	
PR	57 (47.5%)	25 (25.3%)	
SD	44 (36.7%)	45 (45.5%)	
PD	17 (14.2%)	29 (29.3%)	
ORR, n (%)	59 (49.2%)	25 (25.3%)	< 0.001
DCR, n (%)	103 (85.8%)	70 (70.7%)	0.006
Characteristics	IC-A (n=81)	IC+A (n=39)	P value
Best response, n (%)			0.183
SD	35 (43.2%)	9 (23.1%)	
PD	11 (13.6%)	6 (15.4%)	
PR	34 (42%)	23 (59%)	
CR	1 (1.2%)	1 (2.6%)	
ORR, n (%)	35 (43.2%)	24 (61.5%)	0.060
DCR, n (%)	70 (86.4%)	33 (84.6%)	0.791

**Figure 2:**
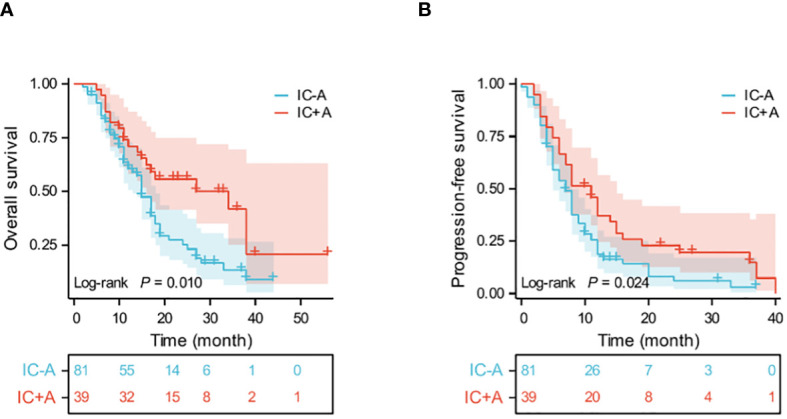
Kaplan-Meier estimates of OS **(A)** and PFS **(B)** in the IC+A and IC-A population; IC+A,PD-1 inhibitor plus chemotherapy with endostatin; IC-A,PD-1 inhibitor plus chemotherapy without endostatin; OS, overall survival; PFS, progression-free survival; Time, month.

Thirty-nine patients in the IC+A group had PFS events in 33 (84.6%) and OS events in 20 (51.2%). Eighty-one patients in the IC-A group had PFS events in 72 (88.8%) and OS events in 56 (69.1%). The median OS was 34 (95% CI: 9.6–58.4) and 15 (95% CI: 13.1–16.9) months (P = 0.01, **Figure 2A**), and the median PFS in the IC+A group and the IC-A group was 11 (95% CI: 7.8–14.2) and 7 (95% CI: 5.5–8.5) months respectively (P = 0.024, **Figure 2B**), and The median PFS and median OS of the IC+A group were longer than those of the IC-A. In the IC+A group, 1 patient (2.6%) achieved CR, 23 patients (59%) achieved PR, 9 patients (23.1%) achieved SD, and 6 patients (15.4%) achieved PD, with an ORR of 61.5% and a DCR of 84.6%. In the IC-A group, 1 patient (1.2%) achieved CR, 34 patients (42%) achieved PR, 35 patients (43.2%) achieved SD, and 11 patients (13.6%) achieved PD, with an ORR of 43.2% and a DCR of 86.4% (**Table 3**). There was no statistical difference in ORR and DCR between the IC+A group and the IC-A group.

### 3.3 Predictors affecting efficacy in the IC ± A group

Multivariate analysis showed that ECOG PS (HR:2.718, 95%CI:1.491–4.953, P=0.001), PD-L1 ≥50% (HR:0.260, 95%CI:0.076–0.889, P=0.032), plus endostatin (HR:0.501, 95%CI:0.289–0.867, P=0.014), chemotherapy cycle ≥4 (HR:0.238, 95%CI: 0.124–0.458, P<0.001), LIPI 0 score (HR:1.672, 95%CI:1.012–2.761, P=0.045) were independent prognostic factors for OS (**Table 4**). In addition, PD-L1 ≥50% (HR:0.392, 95%CI:0.170–0.904, P=0.028), chemotherapy cycle ≥4 (HR:0.312, 95%CI:0.183–0.531, P<0.001), LIPI 0 score (HR:1.821, 95%CI:1.176–2.822, P=0.007) were independent prognostic factors for PFS (**Table 5**).

**Table 4:** Univariate and multivariate Cox analysis of OS in the IC±A group.

Characteristics		Univariate analysis		Multivariate analysis	
		Hazard ratio (95% CI)	P value	Hazard ratio (95% CI)	P value
Age	≤65	Reference			
	>65	1.279 (0.811-2.017)	0.290		
Gender	Female	Reference			
	Male	0.665 (0.288-1.538)	0.340		
ECOG PS	0-1	Reference		Reference	
	2	2.669 (1.551-4.595)	<0.001	2.718 (1.491-4.953)	0.001
Smoking history	No	Reference			
	Yes	0.959 (0.535-1.720)	0.888		
PD-L1	Negative	Reference		Reference	
	1-49%	0.914 (0.498-1.678)	0.771	1.083 (0.582-2.012)	0.802
	≥50%	0.192 (0.057-0.648)	0.008	0.260 (0.076-0.889)	0.032
	Unknown	0.759 (0.431-1.337)	0.340	0.654 (0.364-1.176)	0.156
Clinical stage	IVA	Reference			
	IVB	1.190 (0.665-2.128)	0.558		
Brain metastases	No	Reference			
	Yes	1.489 (0.467-0.750)	0.501		
Liver metastases	No	Reference			
	Yes	0.952 (0.473-1.914)	0.889		
Bone metastases	No	Reference			
	Yes	0.826 (0.475-1.436)	0.497		
Endostatin therapy	No	Reference		Reference	
	Yes	0.524 (0.313-0.878)	0.014	0.501 (0.289-0.867)	0.014
Chemotherapy cycle	2-3	Reference		Reference	
	≥4	0.329 (0.180-0.603)	<0.001	0.238 (0.124-0.458)	<0.001
LIPI	0	Reference		Reference	
	1-2	2.266 (1.408-3.646)	0.001	1.672 (1.012-2.761)	0.045
PD-1 inhibitors	Pembrolizumab	Reference			
	Tislelizumab	1.250 (0.640-2.441)	0.513		
	Sintilimab	1.300 (0.623-2.713)	0.485		
	Camrelizumab	1.296 (0.595-2.821)	0.514		
Chemotherapy	Carboplatin	Reference			
	Cisplatin	0.999 (0.626-1.595)	0.998		

**Table 5:** Univariate and multivariate Cox analysis of PFS in the IC±A group.

Characteristics		Univariate analysis		Multivariate analysis	
		Hazard ratio (95% CI)	P value	Hazard ratio (95% CI)	P value
Age	≤65	Reference			
	>65	1.254 (0.845-1.860)	0.261		
Gender	Female	Reference			
	Male	0.547 (0.264-1.133)	0.105		
ECOG PS	0-1	Reference		Reference	
	2	1.909 (1.131-3.221)	0.015	1.453 (0.812-2.601)	0.208
Smoking history	No	Reference			
	Yes	0.843 (0.516-1.377)	0.496		
PD-L1	Negative	Reference		Reference	
	1-49%	0.606 (0.348-1.056)	0.077	0.623 (0.355-1.094)	0.100
	≥50%	0.289 (0.128-0.650)	0.003	0.392 (0.170-0.904)	0.028
	Unknown	0.627 (0.385-1.021)	0.060	0.552 (0.334-0.913)	0.021
Clinical stage	IVA	Reference			
	IVB	0.924 (0.549-1.556)	0.766		
Brain metastases	No	Reference			
	Yes	1.533 (0.483-4.871)	0.468		
Liver metastases	No	Reference			
	Yes	0.949 (0.518-1.736)	0.864		
Bone metastases	No	Reference			
	Yes	0.752 (0.460-1.227)	0.254		
Endostatin therapy	No	Reference		Reference	
	Yes	0.631 (0.413-0.965)	0.034	0.671 (0.427-1.055)	0.084
Chemotherapy cycle	2-3	Reference		Reference	
	≥4	0.337 (0.202-0.562)	<0.001	0.312 (0.183-0.531)	<0.001
LIPI	0	Reference		Reference	
	1-2	2.079 (1.380-3.133)	<0.001	1.821 (1.176-2.822)	0.007
PD-1 inhibitors	Pembrolizumab	Reference			
	Tislelizumab	1.182 (0.655-2.134)	0.578		
	Sintilimab	1.556 (0.840-2.883)	0.160		
	Camrelizumab	1.507 (0.788-2.881)	0.215		
Chemotherapy	Carboplatin	Reference			
	Cisplatin	0.909 (0.611-1.354)	0.640		

### 3.4 Subgroup analysis

**Figure 3:**
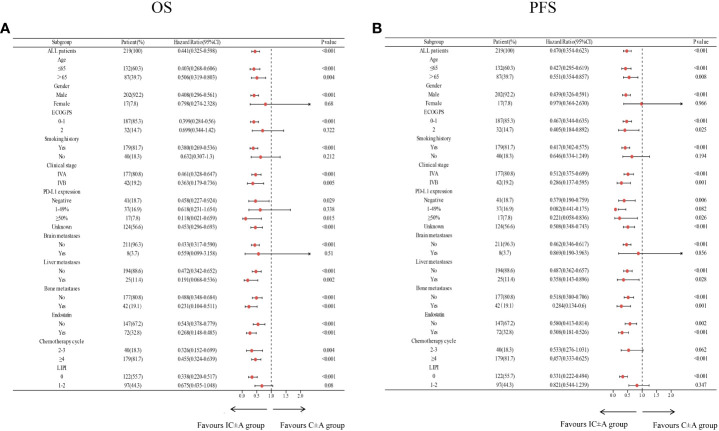
Subgroup analysis of the IC ± A group versus the C ± A group for OS **(A)** and PFS **(B)** based on baseline characteristics; IC ± A,PD-1 inhibitor plus chemotherapy with or without endostatin; C ± A, chemotherapy with or without endostatin; OS, overall survival; PFS, progression-free survival, LIPI, lung immune prognostic index.

Subgroup analysis of IC ± A and C ± A groups showed that IC ± A had an OS benefit in all subgroups except women (P = 0.68), ECOG PS score 2 (P = 0.322), no history of smoking (P = 0.212), PD-L1 expression 1–49% (P = 0.338), brain metastases (P = 0.51), and LIPI score 1–2 (P = 0.08) (**Figure 3A**). Subgroup analysis in the IC ± A and C ± A groups showed that IC ± A had a PFS benefit, except for women (P = 0.966), no history of smoking (P = 0.194), PD-L1 expression 1–49% (P = 0.082), brain metastases (P = 0.856), and LIPI scores 1–2 (P = 0.347) (**Figure 3B**).

**Figure 4:**
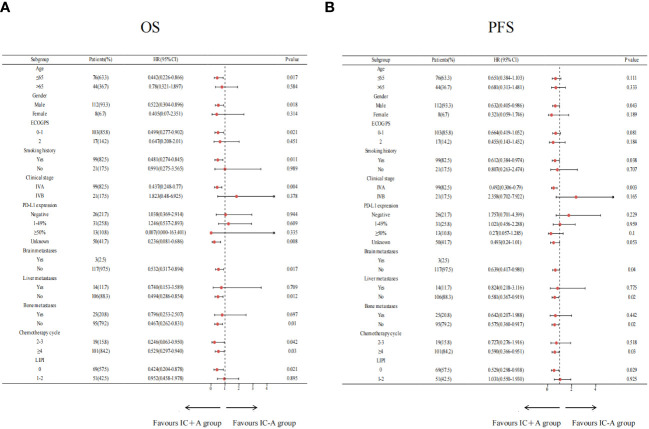
Subgroup analysis of the IC+A group versus the IC-A group for OS **(A)** and PFS **(B)** based on baseline characteristics; IC+A,PD-1 inhibitor plus chemotherapy with endostatin; IC-A,PD-1 inhibitor plus chemotherapy without endostatin; OS, overall survival; PFS, progression-free survival, LIPI, lung immune prognostic index.

The results of the subgroup analysis of IC+A and IC-A are shown in **Figure 4**. Patients with age ≤ 65 (P = 0.017), male (P = 0.018), ECOG PS = 0–1 (P = 0.021), smoking history (P = 0.011), stage IVA (P = 0.004), no brain metastasis (P = 0.017), no liver metastasis (P = 0.012), no bone metastasis (P = 0.01), chemotherapy cycles 2–3 (P = 0.042), chemotherapy cycles ≥ 4 (P = 0.03), and LIPI score of 0 (P = 0.021) had better OS when receiving IC+A treatment (**Figure 4A**). Patients who were male (P = 0.043), history of smoking (P = 0.038), IVA (P = 0.03), no brain metastasis (P = 0.04), no liver metastasis (P = 0.02), no bone metastasis (P = 0.02), chemotherapy cycles ≥4 (P = 0.03), and LIPI score of 0 (P = 0.029) had a better PFS when treated with IC+A (**Figure 4B**).

### 3.5 Safety and adverse events

The adverse events in each group are shown in **Tables 6**, **7**, and the chi-square test for adverse events in each group is shown in **Table 8**. There were 116 (96.6%) any grade AEs and 72 (60%) grade 3–4 AEs in the IC ± A group, while there were 93 (93.9%) any grade AEs and 59 (59.5%) grade 3–4 AEs in the IC-A group. There was no statistical difference in any grade AEs and grade 3–4 AEs between IC ± A and C ± A groups (P > 0.05). In addition, there were 38 (97.4%) and 78 (96.2%) any grade AEs, 24 (61.5%) and 48 (59.2%) grade 3–4 AEs, 14 (35.9%) and 25 (30.9%) any grade irAEs, and 4 (10.2%) and 8 (9.9%) grade 3–4 irAEs in the IC+A group and the IC-A group, respectively. No statistical differences were observed in all AEs, grade 3–4 AEs, any grade irAEs and grade 3–4 irAEs between IC + A and IC-A groups.

**Table 6:** **Adverse events in group IC±A and group C±A**.

	IC±A (n=120)		C±A (n=99)	
Event, n(%)	All	Grade 3-4	All	Grade 3-4
Any	116(96.6)	72(60)	93(93.9)	59(59.5)
Anemia	70(58.3)	25(20.8)	51(51.5)	19(19.1)
Neutropenia	31(25.8)	21(17.5)	29(29.2)	16(16.1)
Thrombocytopenia	20(16.6)	10(8.3)	27(27.2)	10(10.1)
ALT elevation	23(19.1)	7(5.8)	22(22.2)	5(5.1)
Creatinine elevation	14(11.6)	2(1.6)	4(4)	1(1)
Nausea	58(48.3)	6(5)	51(51.5)	4(4)
Decreased appetite	36(30)	3(2.5)	22(22.2)	2(2)
Bronchial or pulmonary infection	40(33.3)	3(2.5)	41(41.4)	3(3)
Rash	21(17.5)	1(0.8)	10(10.1)	1(1)
Vomiting	13(10.8)	1(0.8)	9(9.1)	1(1)
Diarrhea	37(30.8)	4(3.3)	21(21)	3(3)
Pain	25(20.8)	1(0.8)	27(27.2)	1(1)
Insomnia	9(7.5)	1(0.8)	3(3)	1(1)
Immune-related AEs				
Any	39(32.5)	12(10)	/	/
Hypothyroidism	15(12.5)	2(1.6)	/	/
Hyperthyroidism	8(6.7)	1(0.8)	/	/
Pneumonitis	9(7.5)	5(4.1)	/	/
Severe skin reaction	3(2.5)	1(0.8)	/	/
Adrenocortical insufficiency	3(2.5)	1(0.8)	/	/
Hypophysitis	2(1.6)	1(0.8)	/	/
Hepatitis	2(1.6)	2(1.6)	/	/
Nephritis	1(0.8)	1(0.8)	/	/
Colitis	1(0.8)	1(0.8)	/	/
Myocarditis	1(0.8)	1(0.8)	/	/
Arthritis	1(0.8)	1(0.8)	/	/
Pancreatitis	1(0.8)	1(0.8)	/	/
Peripheral neuropathy	1(0.8)	1(0.8)	/	/
Cardiac arrhythmias	1(0.8)	1(0.8)	/	/

/, not available.

**Table 7:** Adverse events in group IC+A and group C-A.

	IC+A (n=39)		IC-A (n=81)	
Event, n (%)	All	Grade 3-4	All	Grade 3-4
Any	38 (97.4)	24 (61.5)	78 (96.2)	48 (59.2)
Anemia	19 (48.7)	10 (25.6)	51 (62.9)	15 (18.5)
Neutropenia	12 (30.7)	9 (23)	19 (23.4)	12 (14.8)
Thrombocytopenia	7 (17.9)	5 (12.8)	13 (16)	5 (6.1)
ALT elevation	8 (20.5)	2 (5.1)	15 (18.5)	5 (6.1)
Creatinine elevation	5 (12.8)	1 (2.5)	9 (11.1)	1 (1.2)
Nausea	20 (51.2)	2 (5.1)	38 (46.9)	4 (4.9)
Decreased appetite	13 (33.3)	1 (2.5)	23 (28.3)	2 (2.4)
Bronchial or pulmonary infection	14 (35.8)	1 (2.5)	26 (32.1)	2 (2.4)
Rash	7 (17.9)	1 (2.5)	14 (17.2)	0
Vomiting	6 (15.3)	0	7 (8.6)	1 (1.2)
Diarrhea	13 (33.3)	1 (2.5)	24 (29.6)	3 (3.7)
Pain	7 (17.9)	0	18 (22.2)	1 (1.2)
Insomnia	3 (7.6)	1 (2.5)	6 (7.4)	0
Immune-related AEs				
Any	14 (35.9)	4 (10.2)	25 (30.9)	8 (9.9)
Hypothyroidism	6 (15.3)	1 (2.5)	9 (11.1)	1 (1.2)
Hyperthyroidism	3 (7.6)	1 (2.5)	5 (6.1)	0
Pneumonitis	4 (10.2)	2 (5.1)	5 (6.1)	3 (3.7)
Severe skin reaction	2 (5.1)	0	1 (1.2)	1 (1.2)
Adrenocortical insufficiency	2 (5.1)	0	1 (1.2)	1 (1.2)
Hypophysitis	1 (2.5)	0	1 (1.2)	1 (1.2)
Hepatitis	1 (2.5)	0	1 (1.2)	1 (1.2)
Nephritis	0	0	1 (1.2)	1 (1.2)
Colitis	0	0	1 (1.2)	1 (1.2)
Myocarditis	0	0	1 (1.2)	1 (1.2)
Arthritis	1 (2.5)	1 (2.5)	0	0
Pancreatitis	0	0	1 (1.2)	1 (1.2)
Peripheral neuropathy	0	0	1 (1.2)	1 (1.2)
Cardiac arrhythmias	0	0	1 (1.2)	1 (1.2)

**Table 8:** **Chi-square test of adverse events in each group**.

	IC±A vs C±A P value		IC+A vs IC-A P value	
Event, n(%)	All	grade 3-4	All	grade 3-4
Any	0.261	0.531	0.608	0.486
Anemia	0.191	0.449	0.1	0.252
Neutropenia	0.337	0.469	0.261	0.194
Thrombocytopenia	0.041	0.412	0.492	0.187
ALT elevation	0.348	0.521	0.488	0.59
Creatinine elevation	0.034	0.572	0.501	0.546
Nausea	0.37	0.499	0.4	0.638
Decreased appetite	0.126	0.59	0.364	0.696
Bronchial or pulmonary infection	0.137	0.564	0.415	0.696
Rash	0.085	0.701	0.558	/
Vomiting	0.423	0.701	0.209	/
Diarrhea	0.073	0.605	0.417	0.608
Pain	0.17	0.701	0.388	/
Insomnia	0.125	0.701	0.609	/
Immune-related AEs				
Any	/	/	0.221	0.59
Hypothyroidism	/	/	0.328	0.546
Hyperthyroidism	/	/	0.514	/
Pneumonitis	/	/	0.325	0.525
Severe skin reaction	/	/	0.246	/
Adrenocortical insufficiency	/	/	0.246	/
Hypophysitis	/	/	0.546	/
Hepatitis	/	/	0.546	/
Nephritis	/	/	/	/
Colitis	/	/	/	/
Myocarditis	/	/	/	/
Arthritis	/	/	/	/
Pancreatitis	/	/	/	/
Peripheral neuropathy	/	/	/	/
Cardiac arrhythmias	/	/	/	/

/, not available.

Incidence rates for all adverse events (AEs) were comparable between the IC+A and IC-A cohorts. Common grade 3–4 AEs for patients undergoing IC+A versus IC-A treatment were as follows: anemia (25.6% vs 18.5%, P = 0.252), neutropenia (23.0% vs 14.8%, P = 0.194), thrombocytopenia (12.8% vs 6.1%, P = 0.187), and ALT elevation (5.1% vs 6.1%, P = 0.59). Regarding immune-related adverse events (irAEs), the prevalences in patients treated with IC+A versus IC-A were hypothyroidism (15.3% vs 11.1%, P = 0.328), hyperthyroidism (7.6% vs 6.1%, P = 0.514), and pneumonia (10.2% vs 6.1%, P = 0.325).

## 4 Discussion

In patients with early-stage LUSC, the combination of PD-1 inhibitors and chemotherapy markedly decreases recurrence rates and improves prognosis. Similarly, for patients with locally advanced unresectable LUSC, this combined therapeutic approach reduces the risk of metastasis and enhances prognosis. However, the majority of patients present with advanced-stage disease at diagnosis, and the median OS for those receiving first-line PD-1 inhibitors and chemotherapy is only 17.2 months (1–3). Endostatin, an antiangiogenic agent, has been demonstrated in preclinical studies to synergistically enhance the impact of PD-1 inhibitors on lung tumor suppression (15). However, clinical evidence is sparse regarding the additional survival benefits conferred by endostatin in patients with stage IV LUSC, who are also receiving PD-1 inhibitors and chemotherapy. Thus, our research represents the initial effort to substantiate the applicability of combining a PD-1 inhibitor with chemotherapy and endostatin for first-line treatment in this patient population. Our findings indicate that the median OS and PFS for this regimen were 34 months and 11 months, respectively. Overall, AEs and irAEs were within acceptable safety margins and manageable. These results imply that incorporating endostatin with PD-1 inhibitors and chemotherapy may offer a novel frirst-line therapeutic option for stage IV LUSC.

In advanced LUSC, the combination of PD-1 inhibitors with chemotherapy has become a standard approach in clinical settings. Data from several clinical trials have established that this combination therapy provides superior ORR, DCR, PFS, and OS compared with chemotherapy alone (3–6). Our study aligns with these findings, demonstrating that, regardless of PD-L1 expression levels, the incorporation of PD-1 inhibitors with chemotherapy confers a greater survival advantage in the first-line management of stage IV LUSC. Despite these improvements, the survival benefit of anti-PD-1 therapy in combination with chemotherapy remains modest for patients with stage IV LUSC.

In 1971, Judah Folkman pioneered the concept of tumor treatment by inhibiting angiogenesis, proposing the theory that tumor proliferation depends on the formation of new blood vessels to supply essential nutrients. He posited that interrupting the tumor’s blood supply could effectively starve the tumor (16). As an angiogenesis inhibitor, endostatin has undergone extensive clinical trials, demonstrating its capacity to target neovascular endothelial cells and suppress tumor growth (17). Notably, one case study reported that the addition of endostatin to PD-1 inhibitors and chemotherapy yielded significant results in treating stage IV LUSC (18). Furthermore, the combination has been attributed with promising efficacy and acceptable safety in the primary treatment of advanced NSCLC (10, 19). Consequently, our retrospective analysis scrutinized the efficacy and safety of PD-1 inhibition with chemotherapy, both with and without the addition of endostatin, in stage IV LUSC treatment. The addition of endostatin was found to markedly enhance OS and PFS in patients. Multivariate Cox regression analysis reinforced the view that endostatin’s synergistic use constitutes an independent prognostic indicator for stage IV LUSC patients undergoing PD-1 inhibitor and chemotherapy treatment. These findings endorse the combination of PD-1 inhibitors, chemotherapy, and endostatin as an emergent first-line treatment modality for stage IV LUSC, meriting adoption in clinical practice.

It is unclear whether the combination of PD-1 inhibitors with chemotherapy and endostatin is effective for all stage IV LUSC patients. Our study conducted a subgroup stratification analysis and found that, in most subgroups—including male patients, smokers, those with an ECOG PS 0–1, stage IVA, and patients without liver, brain, or bone metastases—the OS and PFS were more favorable with the combined treatment of PD-1 inhibitors, chemotherapy, and endostatin than without endostatin. Interestingly, patients with a LIPI score of 0 showed a benefit from the combined treatment, whereas those with LIPI scores of 1–2 did not experience significant advantages from the addition of endostatin. LIPI is assessed on the basis of two hematologic markers, LDH and dNLR, which reflect the systemic immune response to cancer-related inflammation (13, 14, 20). High levels of LDH are associated with cancer cell invasion and metastasis, and patients who have high levels of LDH before immunotherapy have relatively short PFS and OS (21–24). The dNLR reflects the body’s neutrophil levels, which are associated with immunosuppression and promote cancer cell metastasis (25–27). An exploratory pooled analysis of data from 4914 metastatic non-small cell lung cancer patients from 11 randomized multinational clinical trials showed that LIPI is important for predicting the prognosis of patients with metastatic non-small cell lung cancer, and represents a different prognosis by its stratification, which is particularly significant in patients receiving ICIs therapy (14). Consequently, the LIPI score is a vital prognostic marker for immunotherapy and a significant guide for optimizing anti-PD-1 therapy with chemotherapy and endostatin in clinical practice.

Our study is subject to several limitations. Primarily, it is single-centered and retrospective in nature, characterized by a limited sample size, and incomplete detection of PD-L1 expression levels across the study population is a notable deficiency. Moreover, the incidence of survival events was not ubiquitously observed within our cohort, necessitating extended follow-up to amass comprehensive data on survival and adverse events, which would enable a more precise evaluation of the combined efficacy and toxicity of PD-1 inhibitors and chemotherapy with endostatin. Consequently, to elucidate the therapeutic potential of this combination, prospective clinical trials with more extensive participant numbers are indispensable.

## 5 Conclusions

Endostatin, administered concomitantly with chemotherapy and PD-1 inhibitors, yield substantial benefits in OS and PFS and are associated with manageable adverse events. This combination therapy is anticipated to become the preferred initial treatment option for stage IV LUSC, particularly in patients presenting with a LIPI score of 0.

## Data availability statement

The raw data supporting the conclusions of this article will be made available by the authors, without undue reservation.

## Ethics statement

The studies involving humans were approved by Fujian Medical University Union Hospital. The studies were conducted in accordance with the local legislation and institutional requirements. The ethics committee/institutional review board waived the requirement of written informed consent for participation from the participants or the participants’ legal guardians/next of kin because The requirement for informed consent of the patients was waived due to the retrospective nature of this study.

## Author contributions

CL: Conceptualization, Data curation, Methodology, Software, Writing – original draft, Writing – review & editing. YW: Conceptualization, Data curation, Methodology, Software, Writing – original draft, Writing – review & editing. WG: Data curation, Formal analysis, Investigation, Visualization, Writing – review & editing. BD: Data curation, Formal analysis, Investigation, Visualization, Writing – review & editing. NY: Resources, Software, Validation, Writing – review & editing. YZ: Resources, Software, Validation, Writing – review & editing. JZ: Resources, Software, Validation, Writing – review & editing. YH: Data curation, Formal analysis, Investigation, Visualization, Writing – review & editing. JL: Conceptualization, Funding acquisition, Project administration, Supervision, Writing – review & editing.

## References

[1] Chen P Liu Y Wen Y Zhou C. Non-small cell lung cancer in China. Cancer Commun (Lond). (2022) 42:937–70. doi: 10.1002/cac2.12359 PMC9558689 36075878

[2] Lau SCM Pan Y Velcheti V Wong KK. Squamous cell lung cancer: Current landscape and future therapeutic options. Cancer Cell. (2022) 40:1279–93. doi: 10.1016/j.ccell.2022.09.018 36270277

[3] Novello S Kowalski DM Luft A Gümüş M Vicente D Mazières J. Pembrolizumab plus chemotherapy in squamous non-small-cell lung cancer: 5-year update of the phase III KEYNOTE-407 study. J Clin Oncol. (2023) 41:1999–2006. doi: 10.1200/JCO.22.01990 36735893 PMC10082300

[4] Ren S Chen J Xu X Jiang T Cheng Y Chen G. Camrelizumab plus carboplatin and paclitaxel as first-line treatment for advanced squamous NSCLC (CameL-Sq): A phase 3 trial. J Thorac Oncol. (2022) 17:544–57. doi: 10.1016/j.jtho.2021.11.018 34923163

[5] Shi Y Wu L Yu X Xing P Wang Y Zhou J. Sintilimab versus docetaxel as second-line treatment in advanced or metastatic squamous non-small-cell lung cancer: an open-label, randomized controlled phase 3 trial (ORIENT-3). Cancer Commun (Lond). (2022) 42:1314–30. doi: 10.1002/cac2.12385 PMC9759762 36336841

[6] Wang J Lu S Yu X Hu Y Sun Y Wang Z. Tislelizumab plus chemotherapy vs chemotherapy alone as first-line treatment for advanced squamous non-small-cell lung cancer: A phase 3 randomized clinical trial. JAMA Oncol. (2021) 7:709–17. doi: 10.1001/jamaoncol.2021.0366 PMC8017481 33792623

[7] Passaro A Brahmer J Antonia S Mok T Peters S. Managing resistance to immune checkpoint inhibitors in lung cancer: treatment and novel strategies. J Clin Oncol. (2022) 40:598–610. doi: 10.1200/JCO.21.01845 34985992

[8] Zhou K Li S Zhao Y Cheng K. Mechanisms of drug resistance to immune checkpoint inhibitors in non-small cell lung cancer. Front Immunol. (2023) 14:1127071. doi: 10.3389/fimmu.2023.1127071 36845142 PMC9944349

[9] Poluzzi C Iozzo RV Schaefer L. Endostatin and endorepellin: A common route of action for similar angiostatic cancer avengers. Adv Drug Delivery Rev. (2016) 97:156–73. doi: 10.1016/j.addr.2015.10.012 PMC4753091 26518982

[10] Fu S Huang H Shang K Tu G Zhong P Li S. Efficacy and safety of immune checkpoint inhibitors combined with recombinant human endostatin and chemotherapy as the first-line treatment of advanced non-small-cell lung cancer. Future Oncol. (2023) 19:147–58. doi: 10.2217/fon-2022-0861 36779488

[11] Huang H Zhong P Zhu X Fu S Li S Peng S. Immunotherapy combined with rh-endostatin improved clinical outcomes over immunotherapy plus chemotherapy for second-line treatment of advanced NSCLC. Front Oncol. (2023) 13:1137224. doi: 10.3389/fonc.2023.1137224 37035161 PMC10076840

[12] Reckamp KL Redman MW Dragnev KH Minichiello K Villaruz LC Faller B. Phase II randomized study of ramucirumab and pembrolizumab versus standard of care in advanced non-small-cell lung cancer previously treated with immunotherapy-lung-MAP S1800A. J Clin Oncol. (2022) 40:2295–306. doi: 10.1200/JCO.22.00912 PMC9287284 35658002

[13] Sorich MJ Rowland A Karapetis CS Hopkins AM. Evaluation of the lung immune prognostic index for prediction of survival and response in patients treated with atezolizumab for NSCLC: pooled analysis of clinical trials. J Thorac Oncol. (2019) 14:1440–6. doi: 10.1016/j.jtho.2019.04.006 30999110

[14] Kazandjian D Gong Y Keegan P Pazdur R Blumenthal GM. Prognostic value of the lung immune prognostic index for patients treated for metastatic non-small cell lung cancer. JAMA Oncol. (2019) 5:1481–5. doi: 10.1001/jamaoncol.2019.1747 PMC6659150 31343662

[15] Wu J Zhao X Sun Q Jiang Y Zhang W Luo J. Synergic effect of PD-1 blockade and endostar on the PI3K/AKT/mTOR-mediated autophagy and angiogenesis in Lewis lung carcinoma mouse model. BioMed Pharmacother. (2020) 125:109746. doi: 10.1016/j.biopha.2019.109746 32106386

[16] Folkman J. Tumor angiogenesis: therapeutic implications. N Engl J Med. (1971) 285:1182–6. doi: 10.1056/NEJM197111182852108 4938153

[17] Ou-Yang F Lan KL Chen CT Liu JC Weng CL Chou CK. Endostatin-cytosine deaminase fusion protein suppresses tumor growth by targeting neovascular endothelial cells. Cancer Res. (2006) 66:378–84. doi: 10.1158/0008-5472.CAN-05-1578 16397252

[18] Fang Y Sun H Chen Y Jiang N Ji L Shi J. A rapid response of lung squamous cell carcinoma following treatment with sintilimab combined with recombinant humane endostatin injection and nab-paclitaxel in an elderly patient: A case report. Med (Baltimore). (2021) 100:e26801. doi: 10.1097/MD.0000000000026801 PMC8341334 34397833

[19] Pu X Wang Q Liu L Chen B Li K Zhou Y. Rh-endostatin plus camrelizumab and chemotherapy in first-line treatment of advanced non-small cell lung cancer: A multicenter retrospective study. Cancer Med. (2023) 12:7724–33. doi: 10.1002/cam4.5526 PMC10134295 36494905

[20] Mezquita L Auclin E Ferrara R Charrier M Remon J Planchard D. Association of the lung immune prognostic index with immune checkpoint inhibitor outcomes in patients with advanced non-small cell lung cancer. JAMA Oncol. (2018) 4:351–7. doi: 10.1001/jamaoncol.2017.4771 PMC5885829 29327044

[21] Jurisic V Radenkovic S Konjevic G. The actual role of LDH as tumor marker, biochemical and clinical aspects. Adv Exp Med Biol. (2015) 867:115–24. doi: 10.1007/978-94-017-7215-0_8 26530363

[22] Sharma D Singh M Rani R. Role of LDH in tumor glycolysis: Regulation of LDHA by small molecules for cancer therapeutics. Semin Cancer Biol. (2022) 87:184–95. doi: 10.1016/j.semcancer.2022.11.007 36371026

[23] Zhang Z Li Y Yan X Song Q Wang G Hu Y. Pretreatment lactate dehydrogenase may predict outcome of advanced non small-cell lung cancer patients treated with immune checkpoint inhibitors: A meta-analysis. Cancer Med. (2019) 8:1467–73. doi: 10.1002/cam4.2024 PMC6488146 30848091

[24] Adachi Y Tamiya A Taniguchi Y Enomoto T Azuma K Kouno S. Predictive factors for progression-free survival in non-small cell lung cancer patients receiving nivolumab based on performance status. Cancer Med. (2020) 9:1383–91. doi: 10.1002/cam4.2807 PMC7013052 31880861

[25] Ma T Tang Y Wang T Yang Y Zhang Y Wang R. Chronic pulmonary bacterial infection facilitates breast cancer lung metastasis by recruiting tumor-promoting MHCIIhi neutrophils. Signal Transduct Target Ther. (2023) 8:296. doi: 10.1038/s41392-023-01542-0 37563136 PMC10415306

[26] Xiong S Dong L Cheng L. Neutrophils in cancer carcinogenesis and metastasis. J Hematol Oncol. (2021) 14:173. doi: 10.1186/s13045-021-01187-y 34674757 PMC8529570

[27] Nøst TH Alcala K Urbarova I Byrne KS Guida F Sandanger TM. Systemic inflammation markers and cancer incidence in the UK Biobank. Eur J Epidemiol. (2021) 36:841–8. doi: 10.1007/s10654-021-00752-6 PMC8416852 34036468

